# Evidence for age-related vulnerability in dopamine-glutamate projections to the lateral entorhinal cortex

**DOI:** 10.1101/2025.10.06.680552

**Published:** 2025-10-07

**Authors:** Jacquelyn N. Tomaio, Sixtine Fleury, Alexandra Bilder, Jordan Nacimba, Abhilash Lakshman, Yoon Seok Kim, Lief Ericsson Fenno, Charu Ramakrishnan, Karl Deisseroth, Susana Mingote

**Affiliations:** 1 Advanced Science Research Center at the Graduate Center, CUNY, New York, NY, USA.; 2 Department of Psychological and Brain Sciences, Dartmouth College, Hanover, NH, USA.; 3 Gill Institute for Neuroscience and Department of Biology, Indiana University, Bloomington, IN, USA.; 4 Department of Neurology and Neurological Sciences, Stanford University, Stanford, CA 94305, USA.; 5 Stanford University, Stanford, CA, USA.; 6 Department of Psychiatry & Behavioral Sciences, The University of Texas at Austin, Dell Medical School, Austin, Texas, USA.; 7 Department of Bioengineering, Stanford University, Stanford, CA 94305, USA.; 8 Department of Psychiatry and Behavioral Sciences, Stanford University, Stanford, CA, USA.; 9 Howard Hughes Medical Institute, Stanford, CA 94305, USA.

**Keywords:** Aging, dopamine-glutamate co-transmission, lateral entorhinal cortex, ventral tegmental area, memory, INTRSECT

## Abstract

The lateral entorhinal cortex (LEC) is selectively vulnerable to age-related decline and is essential for novelty detection and episodic memory. While DAergic (DAergic) input is known to modulate LEC function, how aging impacts this circuitry remains unclear. Here, we used two viral labeling strategies to investigate projections from the ventral tegmental area (VTA) to the LEC. First, we employed an INTRSECT dual-recombinase approach in TH-Flp::VGLUT2-Cre mice to selectively label dopamine-only (DA-only) and dopamine-glutamate co-releasing (DA-GLU) neurons. Next, we used a DAT-Cre-driven ChR2-YFP strategy to broadly label all DA axons. We found that both DA-only and DA-GLU populations innervate the LEC. With age, we observed a selective reduction in tyrosine hydroxylase (TH) signal within DA axons in the LEC, despite preserved axonal structure as revealed by YFP labeling. VGLUT2 signal within DA-GLU terminals appeared less affected. In the VTA, TH+ neuron density declined with age, with distinct spatial patterns along the anterior-posterior axis. These findings reveal an age-related vulnerability of DAergic projections to the LEC and suggest a circuit-level mechanism may contribute to memory impairments in aging.

## Introduction

Aging can be accompanied by a progressive decline in cognitive abilities, among which episodic memory impairment is especially prominent (Gallagher and Rapp, 1997; Wilson et al., 2006; Alexander et al., 2012). Episodic memory enables the recall of specific events in their contextual framework, and its degradation contributes to many of the challenges faced by older individuals, including an attenuated response to novelty, difficulty discriminating new from familiar experiences and vulnerability to false memories and interference (Norman and Schacter, 1997; Wilson et al., 2006; Reagh et al., 2016). These changes occur in the absence of neurodegeneration and are thought to reflect subtle, circuit-level alterations in temporal lobe regions critical for memory (Rapp and Gallagher, 1996, 1996; Yassa et al., 2010; Reagh and Yassa, 2017; Tran et al., 2019).

Among medial temporal lobe structures, the lateral entorhinal cortex (LEC) emerges as one of the earliest and most selectively vulnerable regions in both normal aging and preclinical Alzheimer’s disease (Khan et al., 2014; Reagh et al., 2018; Tran et al., 2022). Human imaging studies show that reduced LEC activation correlates with impairments in object recognition and discrimination in older adults (Reagh et al, 2018). The LEC is critical involved in novelty detection and item-context integration (Deshmukh and Knierim, 2011; Hunsaker et al., 2013; Tsao et al., 2013; Vandrey et al., 2020; Persson et al., 2022), yet the circuit-level mechanisms underlying its dysfunction and how they drive age-related deficits in recognition and associative memory remain poorly understood.

Dopamine (DA) neurons in the ventral tegmental area (VTA) signal the novelty and salience of incoming stimuli (Schultz, 1998; Bromberg-Martin et al., 2010; Kutlu et al., 2021) and modulate recognition memory (Osorio-Gómez et al., 2022; Fleury et al., 2024). Novelty-induced DA signaling modulates memory by regulating synaptic plasticity in the hippocampus (Huang and Kandel, 1995; Lisman and Otmakhova, 2001; Li et al., 2003; Tsetsenis et al., 2023). However, anatomical studies in mice reveal that DAergic projections from the VTA to the hippocampus are sparse, and that most novelty-induced dopamine release originates from locus coeruleus noradrenergic neurons. (Smith and Greene, 2012; Kempadoo et al., 2016; Takeuchi et al., 2016). In contrast, the LEC receives dense DAergic innervation from the VTA (Swanson, 1982; Mingote et al., 2015), positioning it as a key substrate for novelty-driven DAergic modulation of memory. Indeed, recent work has shown that DAergic signaling in the LEC facilitates associative memory formation by encoding novelty cues. (Lee et al., 2021). It is well established that DAergic signaling is altered in the aged brain (Bäckman et al., 2006), including within temporal lobe regions (Morcom et al., 2010), and that such alterations affect novelty processing in aging (Bunzeck et al., 2007; Chowdhury et al., 2012). How these changes impact the VTA–LEC pathway has not been directly examined.

Over the past two decades, it has become clear that VTA neurons consist of molecularly and functionally distinct subpopulations capable of co-releasing additional neurotransmitters (Tritsch et al., 2012; Eskenazi et al., 2021). Among these, dopamine–glutamate (DA–GLU) neurons represent a unique subpopulation. (Stuber et al., 2010; Root et al., 2016; Mingote et al., 2019; McGovern et al., 2024). These DA-GLU neurons exhibit strong responses to salient stimuli and are contribute to behavioral flexibility and the actions of drugs of abuse (Mingote et al., 2017, 2019; Buck et al., 2021; Eskenazi et al., 2021; McGovern et al., 2024; Warlow et al., 2024). DA– GLU neurons rely on the vesicular glutamate transporter VGLUT2 for glutamate loading and release (Stuber et al., 2010; Wang et al., 2017) and make strong glutamatergic connections to the LEC neurons (Mingote et al., 2015). Co-transmission from VTA dopamine neurons is increasingly recognized as sensitive to aging, with reduced TH and VGLUT2 mRNA expression observed in aged mice (Buck et al., 2025).

We hypothesized that age-related impairments in novelty recognition and associative memory may stem from disruptions in VTA DAergic input to the LEC, particularly within the DA-GLU subpopulation. To test this, we employed the INTRSECT (Fenno et al., 2020) viral labeling approach in TH-FLP::VGLUT2-Cre mice to selectively trace DA-only and DA-GLU neurons in the VTA and quantify their terminal projections to the LEC across lifespans. We combined anatomical mapping with immunohistochemical quantification to assess how aging alters this neuromodulatory circuit. Our findings reveal a selective vulnerability of LEC-projecting DA-GLU neurons, offering new insight into the circuit-specific basis of memory decline in aging.

## Results

### Labeling DA-GLU and DA-only Neurons in the VTA Using an Intersectional Strategy

**Figure 1 -:**
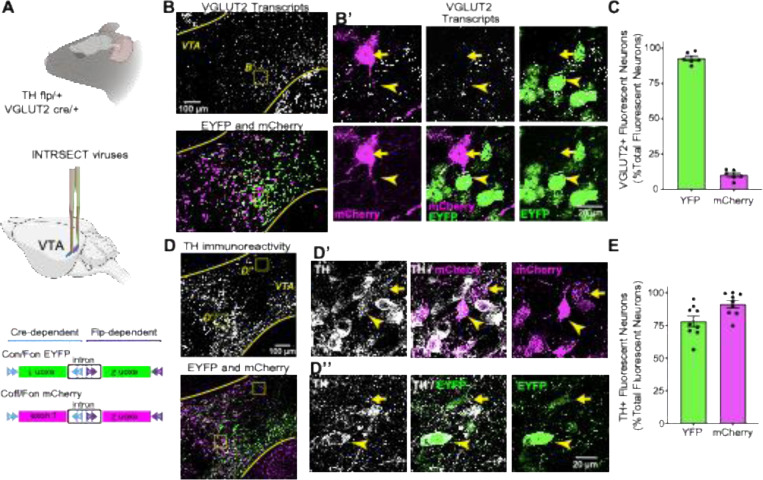
Intersectional labeling distinguishes DA–GLU and DA-only neurons in the VTA using INTRSECT viruses. (**A**) Experimental schematic for double transgenic TH-flp::VGLUT2-cre mice and INTRSECT constructs. Con/Fon drives EYFP in neurons that express both flp and cre (DA–GLU, shown in green) *Figure adapted from* Fenno et al. 2020. Coff/Fon drives mCherry in neurons that express flp but not cre (DA-only, shown in magenta). (**B**) VTA images combining VGLUT2 mRNA *in situ* (top) with EYFP and mCherry immunostaining (bottom); Insert B′ shows EYFP+ somata containing VGLUT2 transcripts and mCherry+ somata containing minimal VGLUT2 signal. (**C**) Quantification of fluorescent neuron subtypes co-expressing VGLUT2 mRNA and EYP (green) or VGLUT2 mRNA and mCherry (magenta), expressed as the percentage of total fluorescently labeled neurons. EYFP+ neurons show high VGLUT2 co-expression. (**D**) Confocal images of VTA sections showing fluorescent labeling of TH+ (top), EYFP+ and mCherry+ neurons (bottom); Insert D’ shows TH immunoreactivity and merged overlays with mCherry; Insert D’’ shows TH immunoreactivity and merged overlays with EYFP. (**E**) Quantification of fluorescent neuron subtypes co-expressing TH protein and EYP (green) or TH protein and mCherry (magenta), expressed as the percentage of total fluorescently labeled neurons. Bar graphs display mean percentages with SEM.

To distinguish DAergic neurons that co-release glutamate (DA-GLU) from those that release only dopamine (DA-only), we used an intersectional viral approach (INTRSECT 2.0; (Fenno et al., 2020) in double transgenic mice (TH-flp/+::VGLUT2-cre/+; Fig. 1A, *top*). These mice express *flp* recombinase under the tyrosine hydroxylase (TH) promoter, marking DA neurons, and *cre* recombinase under the VGLUT2 promoter, marking glutamatergic neurons.

To broadly target the VTA, we injected a mixture of two INTRSECT viruses into both the middle VTA and the lateral VTA/substantia nigra (Fig. 1A, *middle*). The virus AAV8-Con/Fon-EYFP labels neurons that co-express *flp* and *cre*, thereby identifying DA-GLU neurons via EYFP expression. In contrast, AAV8-Coff/Fon-mCherry expresses mCherry in neurons that express *flp* but not *cre*, labeling DA-only neurons (Fig. 1A, *bottom; adapted from* Fenno et al., 2020).

We next assessed how accurately the viruses labeled the subpopulations of DA neurons. To determine glutamatergic identity and specificity, we combined VGLUT2 *in situ* hybridization with EYFP and mCherry immunohistochemistry (Fig. 1B). Confocal photomicrographs (Fig. S1A, *left*) and volume-rendered images (Fig. S1A, *right*) of fluorescence-labeled neurons revealed that 93% ± 1.6% of EYFP+ neurons contained VGLUT2 puncta, confirming successful targeting of DA-GLU neurons. mCherry+ neurons had almost no VGLUT2 signal, consistent with their DA-only identity, although 10% ± 1.4% of mCherry+ neurons showed a single VGLUT2 punctum (Fig. 1C and Fig. S1B).

To evaluate targeting specificity for DAergic neurons, we quantified co-localization with TH immunostaining. We found that 91% ± 2.9% of mCherry+ neurons and 78% ± 4.1% of EYFP+ neurons were TH+ (Fig. 1D,E). The minority of EYFP+ neurons lacking TH immunoreactivity may be part of a previously described subset of VTA neurons expresses TH mRNA but lack detectable TH protein (Yamaguchi et al., 2015). These neurons could be glutamatergic-only (GLU-only) neurons with ectopic EYFP expression, as most EYFP+ neurons also expressed VGLUT2.

Despite a small degree of ectopic EYFP expression in GLU-only neurons, these results demonstrate that the INTRSECT viral strategy is highly effective and selective for labeling DA-GLU neurons with EYFP and DA-only neurons with mCherry, providing a robust tool to distinguish these functionally distinct populations in the VTA and evaluate the effects of aging.

### Distribution of Subpopulations of Dopamine Neurons in the VTA and LEC in Young Mice

**Figure 2.**
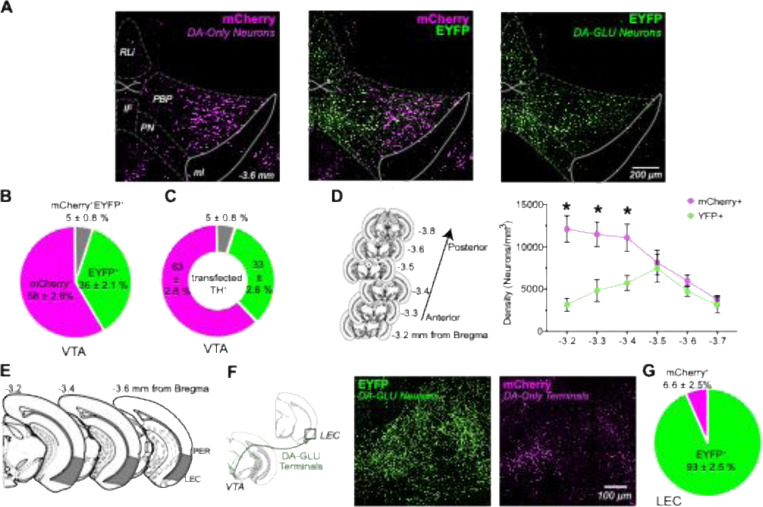
DA–GLU and DA-only neurons exhibit distinct spatial organization in the VTA and differentially innervate the lateral entorhinal cortex in young mice. (**A**) Distribution of INTRSECT labeled VTA neurons expressing EYFP (DA–GLU, green), mCherry (DA-only, magenta), and merge (middle). (**B**) Proportion of EYFP+ (green), mCherry+ (magenta), and co-localized EYFP+/ mCherry+ (gray) neurons in the VTA. (**C**) Proportion of EYFP+/TH+ (green), mCherry+/TH+ (magenta), and EYFP+/ mCherry+/TH+ (gray) neurons in the VTA. (**D**) Schematic of coronal brain sections used for quantification (left); Line plot showing the density (neurons/mm³) of mCherry+ and EYFP+ neurons across the anterior–posterior VTA axis. two-way repeated measures ANOVA; *significant post hoc comparisons, p<0.05. (**E**) Schematic of coronal brain sections from −3.2 mm to −3.6 mm relative to bregma highlighting the lateral entorhinal cortex (LEC) as region of interest and perirhinal cortex (PER). (**F**) Diagram showing the projection from VTA DA–GLU neurons to the LEC (left); Representative image of DA–GLU axons (EYFP+, green) and DA-only axons (mCherry+, magenta) in the LEC. (**G**) Proportion of fluorescent axons in the LEC that are EYFP+ (green) versus mCherry+ (magenta). Graphs show as mean ± SEM.

DA neuron subpopulations show distinct prevalence and spatial distribution patterns within the ventral tegmental area (VTA) (Fig. 2A–C). We found that 36% of all fluorescently labeled cells in the VTA were EYFP+, and when limiting the analysis to TH+ neurons, 33% of transfected TH+ VTA neurons were EYFP+ DA-GLU neurons. This proportion is consistent with prior reports in mice, which estimate DA-GLU neurons to comprise 20–30% of the VTA DA population using intersectional genetic labeling or multiplex *in-situ* hybridization (Yamaguchi et al., 2015; Poulin et al., 2018; Mingote et al., 2019; Eskenazi et al., 2021; Conrad et al., 2024). We observed no differences between males and females; therefore, the data were combined (Fig. S2A–C).

DA-GLU and DA-only neurons also differ in their anatomical distribution. YFP+ DA-GLU neurons are enriched in the interfascicular nucleus (IF), rostral linear nucleus (RLi), and the paranigral part (PBP) of the VTA (Fig. 2A; Conrad et al., 2024). Along the anterior-posterior axis (−3.2 to −3.7 mm from bregma), DA-GLU neurons were predominantly located in the posterior VTA, whereas mCherry+ DA-only neurons were more concentrated in the anterior VTA (Fig. 2D, *right*). A two-way repeated measures ANOVA (2 (cell type) by 6 (distances from bregma, as the within-subject factor)) revealed significant effects of cell type (*F*(1, 8) = 40.36, *p* < .0001, ηp^2^ = .31), distance from bregma (*F*(5, 40) = 6.85, *p* < .0001, ηp^2^ = .28), and their interaction (*F*(5, 40) = 5.58, *p* < .001, ηp^2^ = .24), all in the large effect size range. Post-hoc Bonferroni comparisons indicated that mCherry+ neuron density was significantly greater than EYFP+ at –3.4 mm (*p* = .011), −3.3 mm (*p* = .004), and −3.2 mm (*p* = .001), but not at more posterior levels.

When examining DAergic projections to the lateral entorhinal cortex (LEC), a region known to be especially vulnerable to aging, we found that 93% of labeled axons were EYFP+, indicating that nearly all DAergic input to the LEC originates from DA-GLU neurons (Fig. 2E–G). This was a surprising and novel finding, given the DA-GLU neurons constitute only a minority population within the VTA. Importantly, it builds on our earlier functional connectome work showing robust glutamatergic input from DA neurons to LEC neurons using patch-clamp recordings (Mingote et al., 2015) and together these results validate that DA-GLU neurons play a dominant role in modulating LEC function.

In summary, intersectional strategies reveal that DA-GLU neurons make up roughly one-third of VTA DA neurons, are concentrated in the posterior medial VTA, and provide the majority of DAergic innervation to the LEC.

### Effects of Aging on the Density of DA Neuron Subpopulations within the VTA

The age-related decline of the DAergic system is heterogeneous, affecting some forebrain-projecting DAergic VTA pathways but sparing others (Bäckman et al., 2006; Karalija et al., 2022; Nordin et al., 2022; Johansson et al., 2023). Because subpopulations of VTA dopamine neurons innervate distinct brain regions (Eskenazi et al., 2021), we investigated whether aging differentially impacts these specific subpopulations.

**Figure 3 -:**
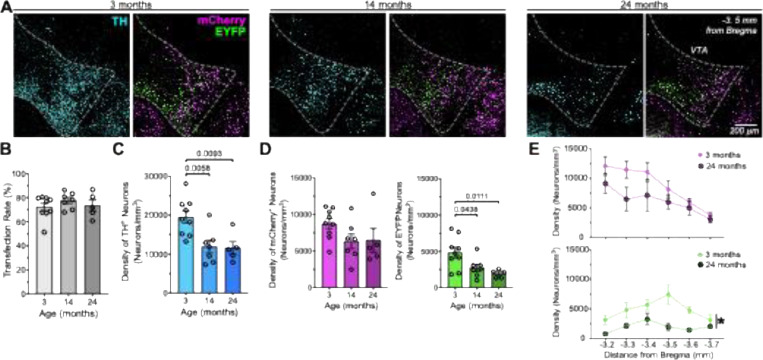
The effects of aging on the density of dopamine neuron subpopulations within the ventral tegmental area. (**A**) Representative unilateral coronal sections through the ventral tegmental area (VTA) at approximately −3.5 mm from bregma in 3- (left), 14- (middle), and 24-month-old (right) mice, showing fluorescent labeling of TH+ (blue), EYFP+ (green), and mCherry+ (magenta) neurons. (**B**) Bar graph showing the transfection rate, calculated as the percentage of TH+ neurons that co-expressed both EYFP and mCherry immunofluorescence, relative to the total number of TH+ VTA neurons, across age groups. (**C**) Bar graph showing TH+ neuron density (neurons/mm^3^) across age groups (one-way ANOVA with Dunnett’s post hoc, *p* values above brackets). (**D**) Bar graph showing mCherry+ (left) and EYFP+ (right) neuron density across age groups (one-way ANOVA, Dunnett’s post hoc, *p* values above brackets). (**E**) Line graphs showing the density of mCherry+ (top) and EYFP+ (bottom) neurons across six anterior– posterior VTA distances (−3.2 to −3.7 mm from bregma) in 3- and 24-month-old mice (two-way repeated measures ANOVA, *main effect of age). Error bars shown as mean ± SEM; Statistics detailed in the text and Methods.

We labeled DA-GLU and DA-only neurons using INTRSECT viral strategies in mice of different ages: 3 months (young, n=9), 14 months (middle, n=7), and 24 months (aged/old, n=5) (Fig. 3A). Double transgenic mice (TH-flp/+::VGLUT2-cre/+) were injected with a combination of Con/Fon-EYFP and Coff/Fon-mCherry viruses one month before reaching the target age group (young: 2 months, *n* = 9; middle-aged: 13 months; aged: 23 months), allowing sufficient time for viral expression before perfusion. A one-month period was allowed for viral expression before the mice were perfused. Age did not affect the transfection rate, which we quantified as the percentage of TH+ neurons in the VTA that co-expressed the transgenes (mCherry and YFP combined) relative to the total number of TH+ VTA neurons, for all ages the average percentage of transfected cells was between 72 to 77% (Fig. 3B). The aging process also did not alter the specificity of the INTRSECT viruses in targeting DA-GLU versus DA-only neurons (Fig. S3 A,B). However, we observed that the overall density of TH+ neurons decreased by ~40% in both middle-aged and aged mice (Fig. 3C). One-way ANOVA revealed an age effect: *F*(2, 18) = 7.91, *p* = .0034, with large effect size (ηp^2^ = .47). Post-hoc Dunnett’s comparisons indicated that density was significantly lower in 14 months (*p* = .011) and 24 months (*p* = .004), relative to 3 months aged mice. To assess whether this age-related decline differentially affected dopamine neuron subpopulations, we separately quantified DA-GLU and DA-only neurons (Fig. 3D). DA-only neurons showed a decreasing trend that did not reach statistical significance (*F*(2, 18) = 1.95, *p* = .171, ηp^2^ = .17, moderate effect size), whereas the density of DA-GLU neurons was significantly reduced with age (*F*(2, 18) = 5.81, *p* = .011, ηp^2^ = .39, large effect size). The density of DA-GLU neurons was significantly lower than in young mice at 14 and 24 months (Post-hoc Dunnett’s comparisons, 14 months (*p* = .044) and 24 months (*p* = .011).

We evaluated how density changes along the anterior–posterior axis by comparing 3- and 24-month-aged mice (Fig. 3E). For the mCherry+ DA-only subpopulation, a two-way repeated measures ANOVA with Age (between-subjects) and Distance from bregma (−3.2 to −3.7 mm, within-subjects) revealed a robust main effect of Distance (*F*(5, 60) = 8.04, *p* < 0.001, ηp^2^ = .455), but no significant main effect of Age (*F*(1, 12) = 3.87, *p* = 0.073, ηp^2^ = .078) and no Age × Distance interaction,(*F*(5, 60) = 0.78, *p* = 0.571, ηp^2^ = .061). In contrast, for the EYFP+ DA-GLU subpopulation, the ANOVA revealed significant main effects of both Distance (*F*(5, 60) = 2.81, *p* = 0.024, ηp^2^ = .25) and Age (*F*(1, 12) = 8.29, *p* = 0.0138, ηp^2^ = .11), while the Age × Distance interaction was not significant (*F*(5, 60) = 1.32, *p* = 0.268, ηp^2^ = .10). These results indicate that although density varies along the anterior–posterior axis for both subpopulations, the Age effect is specific to DA-GLU neurons, reinforcing that the reduction in the density of TH+ neurons at 24 months is primarily driven by loss of TH+ DA-GLU neurons rather than TH+ DA-only neurons.

### Effects of Aging on the Density of DAergic Projections to LEC

**Figure 4 –:**
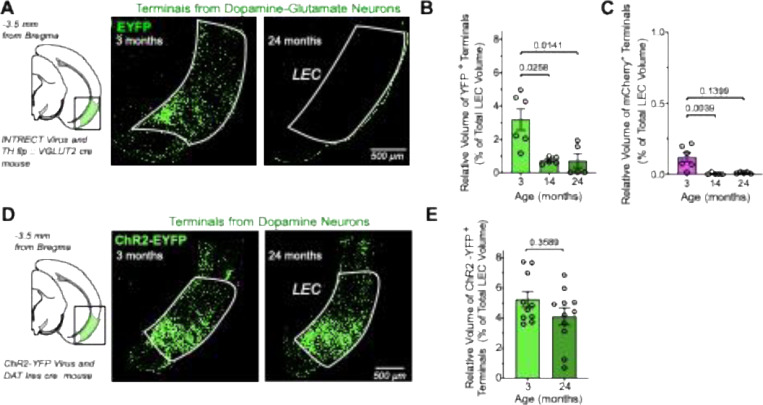
Distinct viral labeling strategies show an age-related reduction in LEC-projecting terminals from VTA dopamine-glutamate (DA-GLU) terminals. (**A**) Schematic of demi-coronal brain section −3.5 mm relative to bregma highlighting the lateral entorhinal cortex (LEC) as region of interest; Representative images showing EYFP-labeled terminals from DA-GLU neurons in the LEC of 3- (left), and 24-month-old (right) TH-FLP::VGLUT2-Cre mice injected with the Cre-On/Flp-On INTRSECT virus. (**B**) Relative volume of EYFP+ terminals in the LEC, expressed as a percentage of total LEC volume. Aged mouse groups showed a significant reduction in EYFP+ terminal volume compared to young mice (unpaired t-test; *p*-values shown on the graph). (**C**) Relative volume of mCherry+ terminals in the LEC, expressed as a percentage of total LEC volume. Aged mouse groups showed a significant reduction in mCherry+ terminal volume compared to young mice (unpaired t-test; *p*-values shown on the graph). (**D**) Schematic of demi-coronal brain section −3.5 mm relative to bregma highlighting the LEC as region of interest; Representative images showing ChR2-EYFP-labeled terminals from DAergic neurons in the LEC of 3- (left), and 24-month-old (right) DAT-Ires-Cre mice injected with the ChR2-EYFP virus. (**E**) Relative volume of ChR2-YFP+ terminals in the LEC, expressed as a percentage of total LEC volume. No significant difference was observed between groups (*p*-value shown on graph). Data are shown as mean ± SEM. Each dot represents one mouse.

Since DA-GLU neurons provide the predominant DAergic input to the LEC, we next asked whether their age-related decline was accompanied by a corresponding reduction in INTRSECT-labeled axons within this region. To quantify terminal density, we performed 3D reconstructions of INTRSECT-labeled axons using volume-rendering software and measured their volume relative to the total volume of the LEC (Fig. 4A). We observed a marked reduction in the density of EYFP-labeled DA-GLU axons, in both middle-aged and aged mice (Fig. 4B). A Kruskal–Wallis test revealed a significant main effect of age (H(2) = 9.26, p = 0.0097), with a large effect size (η^2^H = 0.56). Post-hoc Dunn’s tests confirmed significant reductions in both 14-month (p = 0.0258) and 24-month (p = 0.0141) groups compared to 3-month controls. We also assessed whether the smaller population of mCherry+ axons in the LEC was affected by age and found that expression was significantly reduced at 14 months but not at 24 months (Kruskal–Wallis: H(2) = 9.80, p = 0.0074; Dunn’s tests: 14 months, p = 0.004; 24 months, p = 0.070) (Fig. 4C). Overall, YFP labeling showed substantial losses, averaging ~75% in middle-aged mice and approaching complete loss in some aged animals (Fig. 4A). Of relevance to this study, although DA-GLU neuron density in the VTA declined by only ~40% at 14 months, this was accompanied by nearly a 75% reduction in INTRSECT-labeled axons in the LEC. This disproportionate decline indicates that the LEC is especially vulnerable to early DA-GLU dysfunction.

We further investigated why INTRSECT labeling of DA-GLU neurons and their axons in the LEC declines with age. A recent study reported that TH and VGLUT2 RNA expression in the VTA decreases in aged mice without evidence of cell loss (Buck et al., 2025). We therefore hypothesized that reduced expression of TH or VGLUT2 may underlie the diminished INTRSECT labeling, since this viral strategy depends on the activity of both promoters. To test this, we used an alternative viral strategy in which YFP is expressed under the DAT promoter, independent of TH or VGLUT2 expression status. An AAV-FLEX-ChR2-YFP virus was injected into the VTA of DAT-Ires-Cre mice at 2 or 23 months of age to generate young (3-month-old, *n* = 10) and aged (24-month-old, *n* = 11) cohorts. With this approach, YFP+ DAergic axons in the LEC were clearly observed in both groups (Fig. 4D), and quantification of terminal volume revealed no age-related differences (Fig. 4E). A Mann–Whitney U test confirmed the absence of a significant age effect (U = 68.5, p = 0.36). The small effect size (r = 0.21) further indicates that any difference between groups is minimal. Thus, terminal density in the LEC is largely preserved at 24 months. Taken together, these results argue against cell loss or terminal degeneration and instead suggest that the apparent reduction in INTRSECT labeling reflects decreased TH or VGLUT2 expression with age.

### Decreased Dopamine Synthesis and Glutamate Vesicular Loading with Aging

**Figure 5 –:**
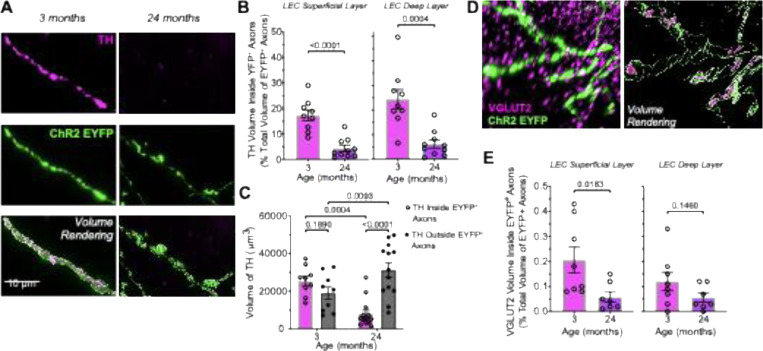
Age-related reduction of TH and VGLUT2 content in LEC-projecting DAergic terminals. (**A**) Representative high-magnification images of axons in the lateral entorhinal cortex (LEC) of 3- (left) and 24-month-old (right) DAT-Ires-Cre mice injected with a ChR2-EYFP. Each column shows TH immunoreactivity (top, magenta), ChR2-EYFP signal (middle, green), and volume rendering (bottom) of merged signals from the same field of view. (**B**) Quantification of TH signal volume within EYFP+ axons, expressed as a percentage of total EYFP+ axon volume, in superficial (left) and deep (right) layers of the LEC. Aged mice exhibited a significant reduction in TH content in both superficial and deep layers (*p*-values shown on graph). (**C**) Absolute volume of TH signal (μm^3^) within (open circles) and outside (closed circles) EYFP+ axons. (**D**) Representative high-resolution image of VGLUT2 puncta (magenta) colocalized with EYFP-labeled axons (green) in the LEC; raw fluorescence image (left); 3D volume rendering of the same region (right). (**E**) Quantification of VGLUT2 signal volume within EYFP+ axons, expressed as a percentage of total EYFP+ axon volume, in superficial (left) and deep (right) LEC. Aged mice showed a significant reduction in VGLUT2 content in the superficial layer (*p* = 0.0183), with no significant difference in the deep layer (*p* = 0.1460). Scale bars, 10 μm.

We investigated whether YFP-labeled DAergic axons in the DAT-Ires-Cre mouse expressed the proteins TH and VGLUT2. To quantify TH expression, we performed volume rendering and generated surfaces of both TH and YFP immunoreactivity (Fig. 5A). TH clusters overlapping by more than 65% with YFP labeling were considered to be inside YFP+ axons, and the volume of TH inside axons was calculated relative to the total volume of YFP+ axons in the LEC. We quantified the superficial and deep layers of the LEC separately, as these layers are functionally distinct (Canto et al., 2008; Witter et al., 2017; Wang et al., 2023) and YFP+ axons were denser in the superficial layer (Fig. S4A). TH expression within YFP+ axons was significantly reduced with age in both layers (Fig. 5A, B; superficial layer: t(17) = 5.51, p = 3.8 × 10^−5^, Cohen’s d = 2.53; deep layer: t(17) = 4.42, p = 3.8 × 10^−4^, Cohen’s d = 2.03), with both declines representing very large effect sizes. In contrast, total TH volume in the LEC, irrespective of overlap with YFP+ axons, showed no age-related decrease (Fig. S4B, C). Since noradrenergic neurons densely innervate the LEC and also express TH (Fallon et al., 1978; Ito and Schuman, 2012; Hernández-Frausto and Vivar, 2024), they may contribute to this apparent preservation of total TH with aging. To test this possibility, we directly compared TH volume inside and outside YFP+ axons. In young mice, TH volume was similar inside and outside, whereas in aged mice TH volume inside axons markedly decreased while TH outside axons increased (Fig. 5C). A two-way ANOVA with Age and TH Location as between-factors revealed a significant interaction (F(1,40) = 22.04, p < 0.0001), a main effect of TH location (F(1,40) = 12.63, p = 0.0010), and no main effect of age (F(1,40) = 0.68, p = 0.41). Pairwise comparisons using Fisher’s LSD test, restricted to four planned comparisons, showed that in young mice TH inside versus outside YFP+ axons was not significantly different (mean diff. = 6,503, p = 0.19), whereas in aged mice TH volume was significantly lower inside than outside axons (mean diff. = −23,221, p < 0.0001). Across ages, TH inside axons was significantly reduced in aged relative to young mice (mean diff. = 17,480, p = 0.0004), and TH outside was significantly higher in aged than in young mice (mean diff. = −12,244, p = 0.0093). These results were confirmed using the more conservative Tukey’s HSD test, which showed no difference between inside and outside in young mice (p = 0.44; d = 0.73, moderate effect), but a robust reduction inside versus outside in aged mice (p < 0.001; d = −2.08, very large effect), a strong age-related reduction inside YFP+ axons (p = 0.002; d = 2.32, very large effect), and a modest but significant age-related increase outside axons (p = 0.044; d = −0.98, large effect).

We then assessed whether VGLUT2 expression is also reduced within YFP-labeled axons. VGLUT2 immunolabeling appeared as densely clustered puncta in the LEC, reflecting expression in both DAergic axons and other glutamatergic inputs, with the latter providing the majority of VGLUT2. The overall volume of VGLUT2 puncta in the LEC did not change with aging (Fig. S4D, E). To quantify VGLUT2 inside YFP+ axons, we acquired images with a confocal microscope equipped with Airyscan super-resolution detection (Wu and Hammer, 2021). Compared to conventional confocal microscopy, Airyscan employs a detector array with pixel reassignment and deconvolution, improving lateral resolution to ~120 nm and enhancing signal-to-noise, thereby allowing a more reliable visualization of densely packed puncta. VGLUT2 puncta overlapping by more than 65% with YFP labeling were considered inside YFP+ axons (Fig. 5D). Using this conservative criterion, we found that in young mice, only ~0.2 % of total VGLUT2 expression in the LEC was localized inside DAergic axons, confirming that the vast majority of VGLUT2 arises from non-DAergic inputs. As with TH, we analyzed superficial and deep layers separately, calculating the volume of VGLUT2 puncta as a percentage of total YFP+ axonal volume. In the superficial layer, VGLUT2 volume was significantly reduced in aged mice (Mann–Whitney U = 48.0, p = 0.024), with large effect sizes (r = 0.60). In contrast, in the deep layer the difference between ages did not reach significance (Mann–Whitney U = 39.0, p = 0.221), although effect sizes suggested a modest shift (r = 0.33).

In conclusion, TH expression was robustly reduced within DAergic axons of the LEC across both superficial and deep layers, whereas TH expression outside YFP+ axons did not decline with age and even showed a modest increase. Given the dense noradrenergic innervation of the LEC, these results suggest that the age-related loss of TH is selective to DAergic inputs and may reduce dopamine availability without altering norepinephrine tone. In parallel, VGLUT2 expression declined with aging in a layer-specific manner, reaching significance only in the superficial LEC. Together, these findings indicate that DA-GLU neurons projecting to the LEC undergo marked age-related deterioration, with a substantial loss of dopamine synthesis capacity and a more moderate, regionally restricted decline in glutamate vesicular loading.

## Discussion

The present study identifies a previously unrecognized, age-related decline in TH and VGLUT2 expression within a subpopulation of DA-GLU neurons projecting to the LEC, uncovering their selective vulnerability to aging.

Using intersectional viral strategies, we selectively labeled DA-GLU and DA-only neurons based on the presence or absence of glutamatergic and DAergic marker expression (VGLUT2 and TH, respectively) (Fenno et al., 2020; McGovern et al., 2024; Warlow et al., 2024). These INTRSECT fluorescent reporters, injected at different ages, faithfully reflected endogenous TH and VGLUT2 expression and captured their age-related decline, providing a robust tool to monitor molecular changes within distinct DAergic subpopulations. INTRSECT-induced EYFP labeling of DA-GLU neurons in the VTA and their axons in the LEC was markedly reduced in middle-aged and aged mice. A ~40% decline in VTA DA-GLU neuron density at middle-aged mice was accompanied by an almost 75% reduction in labeled axons in the LEC, indicating that this DAergic projection is particularly vulnerable to early functional decline. Despite the pronounced loss of INTRSECT-driven signal, there was no evidence of axonal degeneration, as DAergic axons remained detectable in aged mice using an alternative DAT promoter-driven viral strategy. Thus, the reduced INTRSECT labeling reflects decreased TH and VGLUT2 expression rather than structural degeneration. While early studies report DAergic neuron loss with aging by measuring pigmented neurons in the substantia nigra (Ma et al., 1999), more recent work using Nissl and DAPI staining in human and mice (Di Lorenzo Alho et al., 2016; Buck et al., 2025) found that although TH expression declines there is no evidence of overt neurodegeneration in the VTA. Consistent with this, our data reveal no major age-related loss of DAergic axons in the LEC, but rather reduced dopamine synthesis and glutamate vesicular loading. This stands in contrast to Alzheimer’s disease patients and transgenic mouse models, which report DAergic neurodegeneration in the VTA (Nobili et al., 2017; Serra et al., 2018), particularly within its middle portion where DA-GLU neurons are concentrated. Thus, the molecular alterations we identify in DA-GLU neurons during normal aging may represent an early stage of vulnerability that precedes the neurodegenerative phenotypes observed in Alzheimer’s disease.

The decline in TH expression within DAergic axons in the LEC was substantial, with some mice exhibiting reductions of up to 80%. This stands in sharp contrast to the absence of an age-related decline in TH expression outside the labeled DAergic axons, presumably noradrenergic neurons. Noradrenergic neurons originating from the locus coeruleus are themselves vulnerable to aging, showing some noradrenergic terminals loss (for review, Leal and Yassa, 2015). We observed a significant increase in TH signal outside the labeled DAergic terminals in aged mice, which may reflect a compensatory mechanism in response to terminal loss, although further studies are needed to test this possibility. Importantly, DAergic axons diverged from these other sources of TH by showing a unique, age-dependent decrease in synthesis, underscoring the cell type- and pathway-specific nature of aging effects within LEC circuitry. A recent study reported reduced TH mRNA levels in the VTA of aged mice without corresponding decreases in TH protein within the nucleus accumbens core or striatum (Buck et al., 2025). Together with our finding of reduced TH expression in the LEC, this suggests that the VTA neurons exhibiting age-related decreases in TH mRNA may be those projecting to the LEC, highlighting the selective vulnerability of this pathway during normal aging. An additional insight from our study is that in brain regions receiving both DAergic and noradrenergic innervation, overall TH levels may be poor indicators of underlying cellular changes, as opposing alterations in different populations can offset one another, as observed here. TH is the rate-limiting enzyme in dopamine synthesis, and reductions in its expression have been shown to decrease dopamine release (Sulzer et al., 2016). Because TH expression is not completely abolished, our data suggest that dopamine release likely persists but may be compromised in a frequency-dependent manner, with the greatest deficits emerging during high-frequency firing when rapid synthesis is required to sustain release. Dopamine neurons typically exhibit pacemaker-like firing at ~4 Hz but transiently increase their activity in bursts of 20–100 Hz to signal salient events in the environment, such as rewards or novel stimuli (Schultz, 1998; Bromberg-Martin et al., 2010; Farassat et al., 2019). Recordings from DA-GLU neurons have shown that these neurons encode the salience of a stimulus independently of its valence, whether positive and rewarding or negative and aversive (McGovern et al., 2024). Therefore, our findings suggest that this phasic mode of salience signaling may be particularly vulnerable to the age-related reduction in TH expression.

We also observed a reduction in VGLUT2 expression within DAergic axons in the LEC, which became evident only when the superficial and deep layers were analyzed separately. Two key points emerge from these findings. First, the age-related deficit was restricted to the superficial layers (layers II–III), which represent the principal input layers to the hippocampus. DAergic–glutamatergic signaling in this region could therefore play an important role in filtering which cortical inputs to the LEC influence hippocampal activity. We previously showed that DA-GLU neurons form strong excitatory connections onto LEC neurons (Mingote et al., 2015), driving robust depolarization and potentially enhancing their responsiveness to sensory inputs. Thus, age-related reductions in VGLUT2 expression may weaken this filtering or gating function, diminishing the ability of the aged brain to regulate information flow to the hippocampus.

Second, the age-related reduction in glutamate vesicular loading was less pronounced than the decline observed in TH expression, suggesting a potential imbalance in dopamine–glutamate co-transmission. Such an imbalance could promote neurotransmitter switching, in which glutamate is released in the absence of dopamine. Similar switching has been reported in DA-GLU neurons of the hypothalamus in response to alterations in the light–dark cycle (Meng et al., 2018). Studies using CRISPR-mediated deletion of either TH or VGLUT2 in DA-GLU neurons have shown that loss of one protein does not affect the expression of the other. That work suggests that DA-GLU neurons projecting to the nucleus accumbens use dopamine signaling to encode aversive events, whereas glutamate transmission predominates during rewarding events. Whether DA-GLU neurons projecting to the LEC follow a similar logic remains unknown; however, if these neurons shift to a glutamate-only phenotype with age, their functional contribution to behavior could be profoundly altered.

The LEC has been a major focus of research since it was first identified as one of the earliest regions in the memory network to exhibit reduced activity with aging (Khan et al., 2014)and subsequent dysfunction in Alzheimer’s disease (Small and Swanson, 2018). A hallmark behavioral phenotype associated with LEC dysfunction during aging is impaired pattern separation, in which older individuals and aged rodents show a bias toward perceiving novel events as familiar (Burke et al., 2010; Reagh et al., 2018). Studies in young mice have demonstrated that DAergic signaling in the LEC responds selectively to novelty and facilitates the formation of associative memories (Lee et al., 2021). Thus, a reduction in dopamine release with age could diminish novelty-related signaling in the LEC, leading to a bias toward familiarity. Consistent with this idea, recent studies have shown that dopamine signaling in the LEC is disrupted in a mouse model of Alzheimer’s disease and that optogenetic stimulation of these neurons restores associative memory encoding in App-KI mice (Nakagawa et al., 2024). Our findings demonstrate that DA-GLU neurons deteriorate with age, exhibiting deficits in both dopamine synthesis and glutamate vesicular loading. These impairments may trigger a cascade of molecular changes that render the LEC particularly susceptible to age-related dysfunction and contribute to memory decline. In post-mortem tissue from individuals with Parkinson’s disease, there is evidence that melanized dopamine neurons in the substantia nigra lose TH expression before undergoing neurodegeneration (Hirsch et al., 1988). Thus, age-related DAergic deficits in the LEC may heighten its vulnerability to Alzheimer’s disease, a neurodegenerative disorder for which aging remains the principal risk factor.

## Methods

### Experimental Animals.

All procedures were conducted in accordance with National Institutes of Health *Guide for the Care and Use of Laboratory Animals* and approved by the Institutional Animal Care and Use Committee of the City University of New York (CUNY) Advanced Science Research Center (ASRC). Mice were housed under standard barrier conditions with a 12-hour light/dark cycle and ad libitum access to food and water. Male and female mice were used for all experiments. Two transgenic lines were utilized: (1) TH-Flp;VGLUT2-Cre mice for INTRSECT viral experiments and (2) DAT-IRES-Cre mice for ChR2-YFP labeling. Animals were aged to 2–3 months (young), 13–14 months (middle), or 23–24 months (old).

### Stereotactic Surgery

#### INTRSECT Viral Strategy.

To selectively label DA-GLU and DA-only VTA neurons, TH-Flp;VGLUT2-Cre mice were injected with a mixture of AAV8-EF1a-Con/Fon-EYFP-WPRE (labels DA-GLU neurons) and AAV8-EF1a -Coff/Fon-mCherry-WPRE (labels DA-only neurons), following the INTRSECT 2.0 strategy (Fenno et al., 2020). The viruses were kindly donated by Dr. Deisseroth’s lab. Injections were performed at 2, 13, or 23 months of age to allow one month of viral expression prior to tissue collection at 3, 14, or 24 months (n = 9, 7, and 5, respectively). Mice were anesthetized with isoflurane (3% induction, 2% maintenance), and 1 μL of viral solution (1.5 × 10^12^ vg/mL) was pressure-injected into the unilateral VTA and SNc. Stereotaxic coordinates (from bregma) were adjusted by bodyweight: AP −3.0 to −3.4 mm, ML ±0.5 mm, DV −4.1 to −4.5 mm. The pipette remained in place for 5 minutes to minimize backflow.

#### ChR2-YFP Strategy.

To label DAergic axons independent of TH or VGLUT2 promoter activity, DAT-IRES-Cre mice were injected bilaterally in the VTA with AAV5-EF1a-DIO-ChR2(H134R)-EYFP (UNC Lot # AV4313–2B; 1.5 × 10^12^ vg/mL) at either 2 months (n = 10) or 23 months (n = 11). Coordinates and procedure matched the INTRSECT protocol above.

### Immunohistochemistry

#### INTRSECT-Labeled Brains.

Immunohistochemistry (IHC) was performed as previously described (Mingote et al., 2017). Mice were anesthetized (ketamine/xylazine) and perfused with PBS followed by 4% paraformaldehyde (PFA). Brains were post-fixed and cut into 50 μm coronal sections. Sections were blocked in 10% NGS with 0.1% Triton X-100 and incubated with primary antibodies:

- anti-mCherry (rat, 1:5000; Invitrogen, Cat# M11217)
- anti-GFP (chicken, 1:5000; Invitrogen, Cat# A10262)
- anti-VGLUT2 (rabbit, 1:250–1:5000; Synaptic Systems, Cat# 135403)

Secondary antibodies included Alexa Fluor 488, 568, and 647 conjugates. Imaging was performed using a Leica DM6 B epifluorescence microscope or Zeiss LSM 880 confocal with Airyscan Fast.

#### ChR2-YFP Labeled Brains.

Sections were processed similarly but blocked with 10% NGS and 0.5% Triton X-100 in PGBA. Primary antibodies:

- anti-TH (chicken, 1:5000; Aves Labs, Cat# TYH)
- anti-GFP (rabbit, 1:5000; Abcam, Cat# ab6556)

Secondary antibodies were Alexa Fluor 568 and 647 conjugates. Lipofuscin autofluorescence was quenched using cupric sulfate in ammonium acetate (50 mM, pH 5.0) following Schnell et al., 1999. Images were acquired with Leica DM6 B.

#### *In Situ* Hybridization (RNAscope).

*In situ* hybridization was used to detect mRNA transcripts as previously described (Fleury et al., 2024), and combined with IHC following a protocol developed by the manufacturer (ACDBio). RNAscope was performed on 50 μm sections to detect VGLUT2 mRNA, combined with immunostaining for EYFP and mCherry. Sections were subjected to heat and protease digestion per manufacturer protocol. Positive control probes were used to validate mRNA detection.

#### Image Acquisition and Quantification.

3D volume reconstructions and surface renderings were performed using Imaris Bitplane. Regions of interest (VTA, LEC) were defined based on anatomical landmarks from the Allen Brain Atlas. Co-localization of fluorophores was quantified by calculating overlap of >65% volume between markers (e.g., EYFP/TH or EYFP/VGLUT2). Axon density was computed as the volume of labeled terminals normalized to the total LEC volume. All quantifications were performed by at least two independent investigators blinded to age group, and quality checks were conducted to ensure reproducibility and consistency across datasets.

#### Statistical Analyses.

Statistical analyses were performed using GraphPad Prism, with α set at 0.05. Data distributions were assessed using the Shapiro–Wilk test to determine whether parametric or nonparametric tests were appropriate. Between-group comparisons were analyzed using one-way ANOVA, two-way ANOVA, or repeated-measures ANOVA, followed by Dunnett’s or Bonferroni post hoc tests. For non-normally distributed data, Kruskal–Wallis and Mann–Whitney U tests were used with Dunn’s post hoc correction when appropriate. Effect sizes are reported as partial eta squared (ηp^2^), Cohen’s d, or rank-biserial r, depending on the analysis. All statistical tests are reported in the Results and figure legends.

## Supplementary Material

Supplement 1

Supplementary information is available for this paper in the form of figures.
