# Supplementary material for: Evidence for age-related vulnerability in dopamine-glutamate projections to the lateral entorhinal cortex
Source: bioRxiv. 2025 Oct 7:2025.10.06.680552. Preprint. [Version 1] doi: 10.1101/2025.10.06.680552 (PMC12632511; doi:10.1101/2025.10.06.680552)
Supplement: Supplement 1 [file media-1.pdf]

Supplemental Figures 1-4.

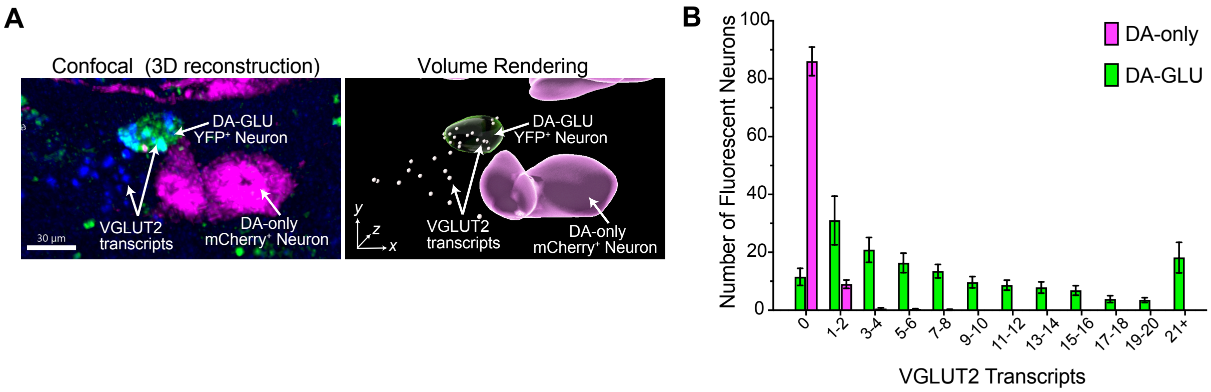

Figure S1 - Validation of recombinase dependence and glutamatergic identity of EYFP+ neurons in the VTA.(**A**) Confocal images (left) and 3D volume renderings (right) of single fluorescently labeled neurons in the VTA showing VGLUT2 transcript puncta (blue) within EYFP+ (green, DA–GLU) and mCherry+ (magenta, DA-only) neurons. Views include orthogonal projections (xy, xz, yz) of each reconstructed cell. Scale bars 30 µm. (**B**) Bar graph quantifying VGLUT2 transcript puncta per neuron in EYFP+ (DA-GLU, green) versus mCherry+ (DA-only, magenta) populations. Statistics not shown in figure.

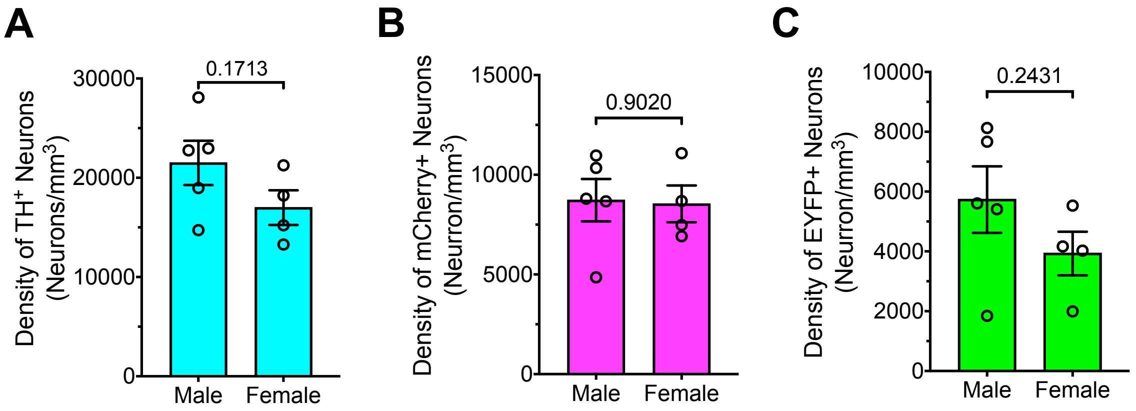

Figure S2 - No sex differences in TH+, mCherry+, or EYFP+ neuron density in the VTA of young mice. Bar graphs comparing the density (neurons/mm³) of TH+ neurons (**A**), mCherry+ neurons (**B**), and EYFP+ neurons (**C**) in male *versus* female young mice. Each bar represents group mean ± SEM. *P*-values are displayed on the plots, indicating no statistically significant differences between sexes for any neuronal population shown.

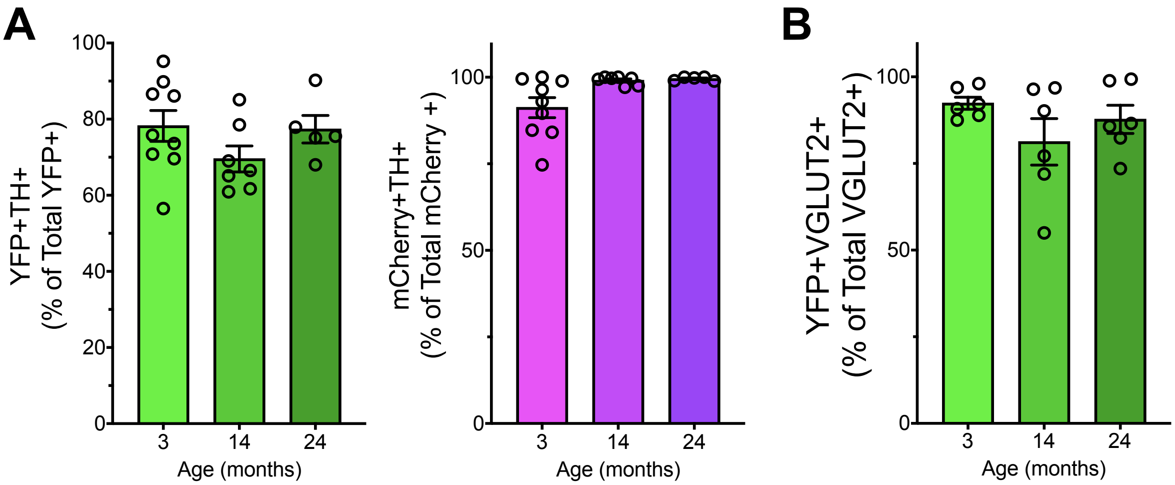

Figure S3 – Age comparison of labeling fidelity in VTA neurons. (**A**) Percentage of YFP+TH+ neurons out of all YFP+ neurons (left) and percentage of mCherry+TH+ neurons out of all mCherry+ neurons (right) at 3, 14, and 24 months. (**B**) Percentage of YFP+VGLUT2+ cells out of all VGLUT2+ cells at 3, 14, and 24 months. Each dot is one animal; bars show mean ± SEM. For each panel, age effects were tested with a one-way ANOVA across the three groups; no significant differences were detected (all $p > 0.05$).

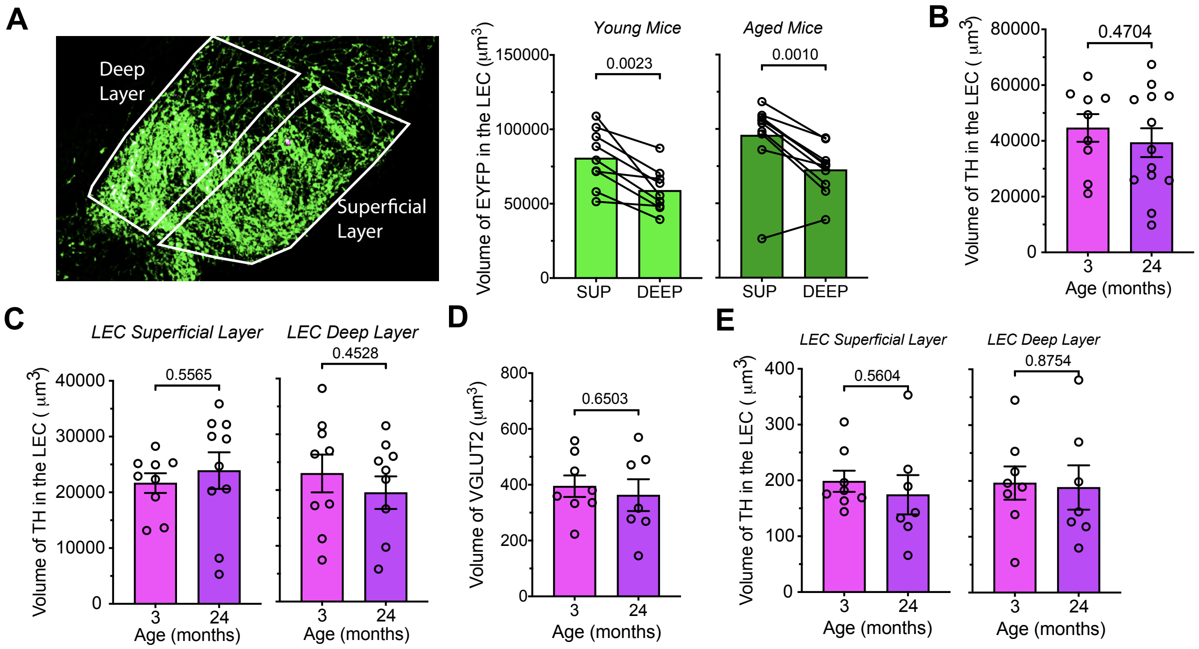

Figure S4 – Analysis of terminal volumes in superficial and deep LEC layers across age. (**A**) Representative image of the LEC showing delineation of superficial and deep layers for volumetric analysis of terminals (left); Total EYFP+ terminal volume (μm³) in superficial and deep LEC layers at 3 and 24 months (right). A significant main effect of age was detected (two-way RM ANOVA: Age × Layer interaction, $F(1, 4) = 9.17$, $p = 0.038$; post hoc Sidak's test: superficial, $p = 0.037$; deep, $p = 0.13$). (**B**) Total TH+ terminal volume (μm³) in the LEC at 3 and 24 months. No significant difference by age (unpaired t-test, $p = 0.19$). (**C**) TH+ volume (μm³) separated by superficial and deep LEC layers across age. No significant Age × Layer interaction or main effects (two-way RM ANOVA, all $p > 0.1$). (**D**) Total VGLUT2+ terminal volume (μm³) in the LEC at 3 and 24 months. No significant difference by age (unpaired t-test, $p = 0.22$). (**E**) VGLUT2+ volume (μm³) in superficial and deep LEC layers at 3 and 24 months. No significant Age × Layer interaction or main effects (two-way RM ANOVA, all $p > 0.1$). Each dot represents an individual animal. Bars show group means ± SEM.
